# The Historical Roots of Visual Analog Scale in Psychology as Revealed by Reference Publication Year Spectroscopy

**DOI:** 10.3389/fnhum.2019.00086

**Published:** 2019-03-12

**Authors:** Andy Wai Kan Yeung, Natalie Sui Miu Wong

**Affiliations:** ^1^Oral and Maxillofacial Radiology, Applied Oral Sciences, Faculty of Dentistry, The University of Hong Kong, Hong Kong, China; ^2^Oral and Maxillofacial Surgery, Faculty of Dentistry, The University of Hong Kong, Hong Kong, China

**Keywords:** citation analysis, psychophysics, psychometric, psychosocial, reference publication year spectroscopy, VAS, visual analog scale

## Abstract

**Background:** Many researchers have been using the visual analog scale (VAS) to acquire psychometric measurements from participants. Several recent studies have consistently pointed to Hayes and Patterson (1921) as the origin of the VAS method. The primary objectives of the current study were to identify the historical root of VAS by cited reference analysis and confirm if it was Hayes and Patterson (1921).

**Methods:** The Web of Science database was searched to identify psychology papers dealing with VAS. The full records and their cited references were extracted and imported into CRExplorer for further analysis. A “reference publication year spectroscopy” (RPYS) was plotted to identify the seminal references.

**Results:** We analyzed 32,569 references cited by 958 articles. There were 21 RPYS peaks ranging from year 1921 to 2007. We were able to identify (Hayes and Patterson, 1921) from the first peak. Furthermore, we were able to identify a total of seven seminal references that are directly relevant to VAS. Two of them were related to “graphic rating method,” three were VAS-validation studies, one was a review on the usage of VAS, and one compared reported results using VAS and Likert scale.

**Conclusions:** Cited reference analysis with a RPYS plot succeeded in identifying and confirming (Hayes and Patterson, 1921) as the origin of VAS. This method has overcome the limitations of conventional citation analysis, namely the issues of being not indexed, not identified by pre-defined search keywords, and not being all-time most cited.

## Introduction

In many experiments and surveys, researchers have to gauge participants' psychometric responses. One of the simplest and commonly used tools for this purpose is the visual analogscale (VAS). Basically, the VAS consists of a continuous horizontal line, usually of 10 cm in printed length, and two descriptive phrases at the two extremities. The scale is commonly ranged from 0 (left, least extreme) to 10 (right, most extreme). Recent studies from various fields, such as cognitive psychology (Kuhlmann et al., 2017), integrative psychology (Sarafian et al., 2018), medicine (Klimek et al., 2017), and public health (Krabbe et al., 2017), have all pointed to the work by Hayes and Patterson in 1921, which introduced the “graphic rating method” (Hayes and Patterson, 1921), as the first scientific description of VAS. The research question here is: is there a systematic method to identify and confirm that (Hayes and Patterson, 1921) is the recognized, first scientific description of the VAS psychometric tool?

The most intuitive method must be to conduct a citation analysis. It can be simply achieved by typing the keywords (i.e., VAS or “visual analog^*^ scale^*^”) into the renowned bibliometric databases such as Web of Science or Scopus, and sorting the search results by citation number. However, we have found that (Hayes and Patterson, 1921) is not indexed in both databases at the time of writing this manuscript. Moreover, they referred the method as graphic rating scale but not VAS. As Web of Science enables users to conduct a cited reference search, we searched and succeeded in finding this publication via this second method. It has been cited in three variants with a total of 144 citations, which was relatively mediocre compared to highly cited VAS publications, such as the one reporting a validation of VAS for pain assessment (Price et al., 1983) with over 1,800 citations.

Therefore, we have performed a cited reference analysis using the CRExplorer software (Thor et al., 2016). It can plot a “reference publication year spectroscopy” (RPYS), which shows a waveform along the timeline, and the waveform illustrates in which years the more cited references (compared to preceding and succeeding years) were published (Marx and Bornmann, 2014; Marx et al., 2014; Wray and Bornmann, 2015; Yeung, 2017; Yeung et al., 2019). The primary aims of the current study were to identify the historical root of VAS by RPYS and confirm if it was Hayes and Patterson (1921). The secondary aim was to identify the representative publications at the subsequent RPYS peaks.

## Materials and Methods

The Web of Science Core Collection online database was accessed on 18 October 2018. A search was performed using the following strategy: TOPIC = (visual analog^*^ scale^*^). The topic search did not include the abbreviation VAS because this abbreviation has multiple meanings such as vibroacoustic stimulation and value-added service. The search was restricted to publications in journals classified by Web of Science as in any fields of psychology, namely “Psychology,” “Psychology, Applied,” “Psychology, Biological,” “Psychology, Clinical,” “Psychology, Developmental,” “Psychology, Educational,” “Psychology, Experimental,” “Psychology, Mathematical,” “Psychology, Multidisciplinary,” “Psychology, Psychoanalysis,” or “Psychology, Social.” No additional restriction was placed, such as on the publication year, or language. The search yielded 958 articles, which collectively had 32,569 cited references.

The full record and cited references of these 958 articles were imported into the CRExplorer software. It was developed to identify publications, within a pre-defined body of literature, that have been most frequently referenced (Marx et al., 2014; Wray and Bornmann, 2015). The RPYS plot has two components: a bar chart that illustrates the raw frequency of cited references published in each year, and a spectrogram that shows positive and negative peaks that indicate years when the citation count has deviated from its 5 years median. For example, references published in 2002–2006 were cited 1,328, 1,364, 1,413, 1,249, and 1,268 times, respectively. The 5 years median citation count was 1,328. Therefore, references published in 2004, being cited 1,413 times, were cited 85 times more than its 5 years median and produced a positive peak with a magnitude of 85. Positive peaks indicated higher-than-average citation count received by references published in those years. We only considered positive peaks with a magnitude of at least 5. For the positive peaks with a magnitude < 50, we evaluated the references with >10% contributions to the peak. For the peaks with a magnitude >50, we also evaluated the references with >10% contributions to the peak, or the top two contributing references if none of them had >10% contribution. These thresholds were set to focus on more important cited references, similar to a previous study that excluded references with <10% contributions to a peak (Bornmann et al., 2018). To provide additional insight, the N_TOP10 indicator was checked for the seminal references identified. This indicator identifies in how many years a reference was a top 10% cited reference, within the dataset. The dataset (VAS.cre) is supplied as a zipped Supplementary Data Sheet that can be unzipped and imported into CRExplorer.

## Results

### Distribution of the Positive Peaks

**Figure 1:**
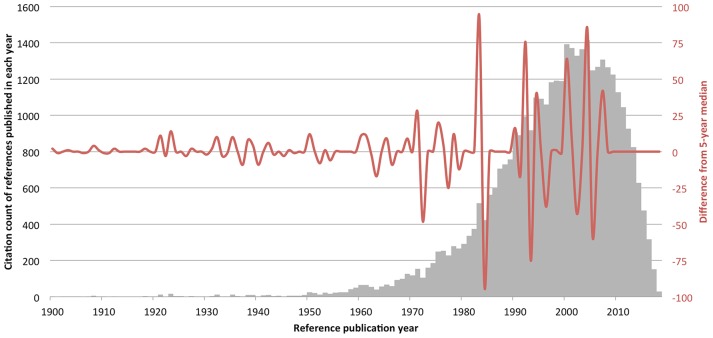
Results from reference publication year spectroscopy (RPYS). The reference lists of the 958 psychology publications concerning visual analog scale were analyzed by CRExplorer. References were sorted by publication year (x-axis), and the citation counts received by each reference published in the same year were summated (gray, left y-axis). The spectrogram has plotted the difference in annual citation count from its 5 years median (red, right y-axis).

There were 21 peaks ranging from year 1921 to 2007 (Figure 1). The largest peaks were in the 1980s−2010s. The seminal references that fulfilled our defined criteria were listed in Table 1. They were among the top 10% cited references for 2–20 years. By this method, we were able to identify (Hayes and Patterson, 1921) from the first peak. Furthermore, we were able to identify a total of seven seminal references that are directly relevant to VAS. Two of them were related to “graphic rating method,” three were VAS-validation studies, one was a review on the usage of VAS, and one compared reported results using VAS and Likert scale. The other seminal references not directly relevant to VAS were mostly studies reporting results with ordinal scales, such as Likert scale.

**Table 1:** Seminal references identified by reference publication year spectroscopy (RPYS).

**Year**	**References**	**Times cited by 958 articles**	**% contributions to the peak**	**Times being a top 10% cited reference of a year, within the dataset**	**Relevance to visual analog scale (VAS)**
1921	Hayes and Patterson, 1921	11	91.7	5	Described graphic rating scale
1923	Freyd, 1923	7	41.2	6	Further described graphic rating scale
1932	Likert, 1932	8	66.7	4	(Irrelevant) Introduced Likert scale
1935	Stroop, 1935	9	75.0	7	(Irrelevant) Introduced the Stroop task
1938	Jacobson, 1938	4	36.4	4	(Irrelevant) Described progressive relaxation
1942	Hayman, 1942	3	27.3	2	(Irrelevant) Described a test to measure intellectual impairment
1960	Hamilton, 1960	29	44.6	16	(Irrelevant) Described an ordinal scale to assess depression
1961	Beck et al., 1961	33	50.8	17	(Irrelevant) Described an ordinal scale to assess depression
1965	Zung, 1965	9	13.4	9	(Irrelevant) Described an ordinal scale to assess depression
1969	Aitken, 1969	25	19.8	20	Review paper on the use of VAS; advocated the use of 100 mm-line VAS
1983	Zigmond and Snaith, 1983	31	6.0	17	(Irrelevant) Described an ordinal scale to assess depression and anxiety
	Price et al., 1983	29	5.6	20	Validated VAS for measuring pain intensity
1992	Cohen, 1992	16	1.6	10	(Irrelevant) Defined the magnitude of effect sizes for common interpretations
	Hunt and McKenna, 1992	6	0.6	4	(Irrelevant) Described a dichotomous scale to assess depression
2000	Flint et al., 2000	13	0.9	8	Validated VAS for measuring appetite
	Sayette et al., 2000	6	0.4	6	(Irrelevant) Reviewed magnitude estimation on craving
2004	de Boer et al., 2004	10	0.7	7	Validated single VAS score for measuring quality of life
	Flynn et al., 2004	9	0.6	8	Advocated Likert scale over VAS for measuring functional dimensions of coping

## Discussion

Traditional bibliometric analyses have focused on the publication and citation counts of a body of literature identified by a refined search strategy (Yeung et al., 2017). By doing so, it would be impossible for us to identify (Hayes and Patterson, 1921) due to its relatively small number of citations received and its referral to the method as graphic rating scale instead of VAS. It is understandable, since the use of VAS scale has become a routine practice that researchers may not necessarily cite the original paper to substantiate their use of the method. However, bibliometricians and librarian scientists have recently attempted to examine the cited references of selected publications in order to analyse the data from another angle (Marx et al., 2014; Comins and Leydesdorff, 2016). Marx and Bornmann have pointed out that the most crucial edge of this approach over the conventional citation analysis is its ability to identify the historical roots of a pre-defined body of literature, which can be conceptually heterogeneous and thus not found by the search keywords, or may be highly cited compared to other references published during those years but not all-time highly cited (Marx and Bornmann, 2016). In our case, we succeeded in identifying the not-so-highly-cited (Hayes and Patterson, 1921) as the first key reference and scientific description of the graphic rating method, which can be considered as the origin of VAS. According to Web of Science, it was cited by 144 publications only. From our cited reference analysis, it was cited by 11 of the 958 publications analyzed (1.1%). These figures were not outstanding. However, the 958 publications have only cited a total of 12 times to all references published in 1921, and that (Hayes and Patterson, 1921) was accountable for 11 (91.7%) of them. Moreover, the RPYS has shown that references published in 1920 and 1922 have collectively received one and zero citation, respectively. These findings have highlighted the relative importance of Hayes and Patterson (1921).

The current analysis has certain limitations. One limitation could be the missing abbreviation of VAS in the search query. Another limitation might be the coverage of the employed database, which did not allow the analysis of publications not indexed.

## Conclusions

The validity of VAS has been continuously assessed by researchers in different fields, such as for the evaluation of pain, mood, and craving [c.f. Wewers and Lowe (1990)]. Surely, no psychometric scale would fit all research purposes, but it is expected that VAS will continue to flourish due to its simplicity in administration. The seminal works by Hayes, Patterson and Freyd may not be well cited by the scientific literature, but the results from the current study have appreciated and reaffirmed their contributions to this very important aspect of psychology. The authors would encourage colleagues to use similar methodology to probe into the origin of a particular method or technique of interest, whenever they are in doubt.

## Data Availability

All datasets generated for this study are included in the manuscript and/or the supplementary files.

## Author Contributions

All authors listed have made a substantial, direct and intellectual contribution to the work, and approved it for publication.

### Conflict of Interest Statement

The authors declare that the research was conducted in the absence of any commercial or financial relationships that could be construed as a potential conflict of interest.
